# Predators among protectors: overcoming power abuse during humanitarian crisis through effective humanitarian diplomacy and a gender-transformative approach

**DOI:** 10.3934/publichealth.2021015

**Published:** 2021-02-26

**Authors:** Sumbal Javed, Vijay Kumar Chattu, Hamid Allahverdipour

**Affiliations:** 1 School of Public Health, The University of Hong Kong, Hong Kong; 2 Department of Medicine, Faculty of Medicine, University of Toronto, Toronto, ON M5G 2C4, Canada; 3 Global Institute of Public Health, Thiruvananthapuram, Kerala, India; 4 Research Center of Psychiatry and Behavioral Sciences & Department of Health Education and Promotion, Tabriz University of Medical Sciences, Tabriz, 14711, Iran

**Keywords:** sexual exploitation and abuse, humanitarian diplomacy, gender, international organizations, United Nations

## Abstract

Sexual exploitation and abuse (SEA) is one of the most depraved crimes against humanity. When carried out by peacekeepers and humanitarian aid workers, it depicts a catastrophic failure of protection bringing harm to the very people the United Nations and international organizations vow to protect. This paper has highlighted the various allegations and incidents of SEA repeatedly happening in conflict-affected countries. Allegations of SEA have since surfaced related to operations in Bosnia, Cambodia, Somalia, Sierra Leone, Liberia, Kosovo, Cote d'Ivoire, Haiti, Sudan, Guinea and the Democratic Republic of Congo. The symptoms of abuse survivors generally resemble those of Post-Traumatic Stress Disorder, and SEA has significant health consequences and poses a severe threat to public health advancement. Based on the literature review, we propose that international and humanitarian organizations must ensure that these offences do not happen in the future by taking appropriate measures. These organizations must prioritize rigorous training on gender equality and values and include a basic mandatory test on gender equality before joining humanitarian missions. Since humanitarian diplomacy encompasses actions carried out by the humanitarian organizations to acquire space from military and political authorities within to function with integrity, we emphasize that strengthening humanitarian diplomacy can play a pivotal role to train the humanitarian workforce on best practices to reduce SEA. Besides, we further propose that women should be allowed to lead from the front; otherwise, true gender equality and issues relevant to gender, including SEA, will be challenging to attain.

## 1. Introduction

Sexual exploitation and abuse (SEA) is one of the most depraved crimes against humanity. When carried out by peacekeepers and humanitarian aid workers, it depicts a catastrophic failure of protection bringing harm to the very people the United Nations (UN) and international organizations vow to protect. It jeopardizes much needed humanitarian aid missions and undermines the legitimacy and credibility of all parties involved. SEA is also a violation of universally recognized international legal norms and standards [1],[2]. The UN defines sexual exploitation as “any actual or attempted abuse of a position of vulnerability, differential power, or trust, for sexual purposes, including, but not limited to, profiting monetarily, socially or politically from the sexual exploitation of another” and sexual abuse as the actual or threatened physical intrusion of a sexual nature, whether by force or under unequal or coercive conditions [3]. This illuminates that the UN considers the SEA as actions that include peacekeepers and humanitarian aid workers involved in transactional sex as sexual violence.

SEA received serious attention in both academic and policy circles when UN Security Council passed Resolution 1325 on Women, Peace and Security (UNSCR1325) in the year 2000 and even more so after The Zeid report was released to the UN Secretary-General [4]–[6]. It garnered much-needed attention and commitment to preventing SEA in missions under UN auspices. The Zeid report presented detailed SEA allegations by peacekeepers in the Congo and revealed how operatives were obstructing investigations. The report further revealed the UN's inefficiency in discipline and control of the peacekeepers in the field due to technical issues and inter alia legal. SEA has received more consistent attention over the last two decades from both academics and organizers [5],[6].

## 2. Power abuse in the aid sector

UN has pledged “zero tolerance” and has developed policies to eradicate SEA, but despite the efforts, it remains wide-spread and continues to be underreported [7],[8]. In the early 1990s, a sex trafficking network connected to UN peacekeepers was exposed in Bosnia and allegations of SEA have since surfaced related to operations in Cambodia, Somalia, Sierra Leone, Liberia, Kosovo, Cote d'Ivoire, Haiti, Sudan and Guinea [6],[9],[10]. Most recently, more than fifty women have accused UN personnel and humanitarian aid workers of SEA during the Ebola outbreak from 2018–2020. The large-scale Ebola operation effectuated thousands of jobs being filled by locals in the poverty-stricken region. As men were holding most of the decision-making positions, it led to the exploitation of women who were keen on getting well-paid jobs. Workers hired locally and internationally were employed on temporary contracts as consultants. According to a WHO employee, “Knowing the poverty of the population, many consultants amused themselves by using sexual blackmail for hiring”. About thirty women informed the investigators that they were sexually exploited or abused by workers identifying themselves as employees of WHO, Médecins Sans Frontières (MSF), UNICEF, Oxfam, ALIMA, World Vision and the UN's IOM migration agency [11],[12].

Further, A women informed of two different incidences when she was offered money for sex; one of the men said he worked for WHO, and another man told her he worked for UNICEF. “Why would you even ask if I reported it?”, “I was terrified”. I felt disgusting. I haven't even told my mother about this [11]. Few of the locals and aid reporters aware of SEA incidents ever report it; even fewer women believed that they could get justice, as the sex-for-jobs practice was so wide-spread. Some of them suggested more vital programmes and policies to prevent and report SEA instead of taking an adversarial approach as whistleblowers [11]–[13]. Humanitarian aid sector experts have blamed these failures on male-dominated operations and lack of funding to tackle SEA; power and income inequalities that open the door to offenders; and limited communication with the locals, problems which have taken place in numerous emergency responses [14],[15].

According to Alina Potts, a research scientist at the Global Women's Institute at George Washington University, COVID-19 has put communities at a higher risk. Funding is often limited, and most of it is moving away from services like gender-based violence to COVID-19 pandemic response. Safe spaces for women to access services and receive confidential support have closed, and the pandemic is “exacerbating these existing power imbalances”, making it more difficult to report abuse. The pandemic is impacting efforts to address SEA within the aid sector. She further stated that “We have communities that have maybe less access to aid—it's harder for them to travel and to move around. There's a lot of fear. We can see a real vacuum where much humanitarian staff no longer go into communities. People can then exploit that vacuum and say, “I can get you access; I can get you the assistance if you have a relationship with me” [2],[16],[17]. UN Secretary-General António Guterres has asked for an investigation. The UN and non-governmental organization (NGO) have repeatedly affirmed to improve efforts to combat SEA, but the problem continues to persist [11],[17].

## 3. Public health implications

In Haiti, the 2010 cholera epidemic was one of the largest cholera epidemics ever recorded. Almost 10 000 people have died due to the introduction of cholera by UN peacekeepers; it is a tragic reminder that peacekeepers can be agents of disease spread [18],[19]. The relatively large number of UN missions in Africa, and the prevalence of AIDS, the correlation between transactional sex and the spread of illness makes SEA a critical problem for missions. SEA can impact local development and relations between local women and men; it can increase the risk of spreading sexually transmitted infections, HIV/AIDS and unwanted pregnancies [5],[6],[20].

If the aid workers have multiple partners and proper precautions have not been taken when engaged in transactional sex or abuse, it can be a health risk for women and their partners. The local public health infrastructure at times is not well developed to handle these health issues. Further, the symptoms of abuse survivors generally resemble those of Post-Traumatic Stress Disorder (PTSD). SEA has significant health consequences and poses a severe threat to public health advancements [20]–[22].

## 4. Humanitarian aid a necessity

Despite the shortcomings such as SEA, humanitarian aid is a necessity amid the rising humanitarian crisis. In 2010 about 53 million people relied on humanitarian aid, which increased to 136 million in 2018. More than 68 million people have had to flee their homes, most extensive of this numbers coming from the crisis in Sudan, South Sudan, Somalia, Yemen and Syria, all of which were due to the violent conflict. Poverty combined with geopolitical instability and protracted crises, rapid population growth and climate change has all been a recipe for disaster. These issues have led to the far more complex humanitarian crisis, which lasts longer, on average, nearly ten years and has impacted a much bigger population than in the past. The humanitarian crisis is not an isolated phenomenon as often a regional occurrence will have a global impact. In a dire and dramatic situation, humanitarian aid is not only welcome when one's survival is at stake, but it is a necessity that cannot be turned down. Regardless of its origin, sometimes it is the only way to meet the most pressing needs [23]–[25].

## 5. Humanitarian diplomacy

Humanitarian diplomacy is deeply rooted in the history of humanitarian aid. It goes as far back as the nineteenth century, drawing its raison d'être from the efforts and contributions made by the humanitarian aid workers not only internationally but also nationally and locally to have access to victims at all times [25]. Humanitarian diplomacy has been defined as the concept that encompasses actions carried out by humanitarian organizations to acquire space from military and political authorities to function with integrity. These activities consist of efforts required to arrange the humanitarian organization's presence in a given country, advance recognition and respect for international law, and advocate for various humanitarian objectives. Humanitarian diplomacy, like other forms of diplomacy, is multicultural and multiform [24],[26]. Humanitarian diplomacy is more focused on persuading decision-makers and opinion leaders to act, at all times, in the interest of vulnerable people and with full respect for our fundamental principles. Humanitarian workforce globalization has resulted in a multicultural approach and methods of practicing humanitarian diplomacy, which given the vast array of context and location of crisis, is indispensable [27].

Contributing-country gender-equality performance matters are in line with the concept that a proclivity for SEA misdeeds is a learned behaviour. Patriarchal beliefs are not fixed and can be changed. Thus, such tendencies can be unlearned by building interpersonal and psychological skills of the humanitarian workforce as part of their training to dissuade SEA while developing their capacities to engage in humanitarian diplomacy in various crises and different parts of the world. While having a greater representation of women can aid in reducing SEA propensity, introducing policies that displace the entire burden of SEA onto the shoulders of one group is likely to have limited effectiveness in addressing the fundamental roots of the issues. A more comprehensive approach towards fostering gender equality among the humanitarian workforce, including peacekeepers, is imperative. Through humanitarian diplomacy, advocating for institutionalizing gender equality, professionalizing training and gender mainstreaming as part of participating in humanitarian aid operations will yield positive dividends concerning SEA [6],[20],[21].

Mastering the appropriate international human right laws and regulations concerning SEA should form part of the humanitarian workforce training. Understanding humanitarian law and human rights are fundamental to avert human right violations, including SEA. Peacekeeping training centres should ensure to include human right-specific courses which, in light of rising cases of SEA, adequately address human rights issues while making greater use of human rights experts [28],[29].

Operational, technical tasks, logistical and insecurity inherent in crises are some of the areas in which humanitarian aid workers receive their training. There are several prerequisites for humanitarian aid workers, including proficiency in languages, driving, computer literacy and achieving a certain rank. The increasing SEA offences make it crucial to include rigorous training on gender equality values and to include a basic test on gender equality mandatory before joining humanitarian missions. Humanitarian diplomacy can play a pivotal role to train the humanitarian workforce on best practices to reduce SEA [30]–[32].

## 6. A gender-transformative approach to SEA

The international humanitarian aid system is not equipped adequately for the humanitarian crisis challenges posed by the twenty-first century. Initiatives have been taken to reform the global humanitarian aid system. While noteworthy, these initiatives will not get to the crux of the problem until the sector addresses the dynamic of gender, power and entitlement that impact the choices made by some aid workers to abuse and exploit the locals, which means fewer cases are reported and then investigated. There is a need to ensure that the leadership is committed and accountable for safeguarding policies for SEA prevention and understanding that power and gender are major driving forces of dereliction. And they need to move away from understanding sexual misconduct as something individual staff perpetrate but see it as the product of the complex environments in which they work and address the causal and contextual factors that give rise to them [33],[34].

To better understand and prevent SEA, a situational crime prevention perspective can be applied, which focuses on the aspects of the immediate environment that encourage or permits crime to occur. It demonstrates that whole behaviour is the consequence of an interaction between the actor's characteristics and the circumstances in which an act is performed. The environment plays a vital role in influencing and shaping the action and is not merely a passive backdrop. Therefore, the probability of crime occurrence varies depending on both the person's criminal disposition and the crime facilitating nature of the backdrop. Situational crime prevention is about creating a safe environment that can facilitate SEA prevention [35].

Women in humanitarian crisis, i.e., armed conflict, natural disasters and armed conflict, have a right under international law to be a part of and are aware of the decisions affecting their lives. These rights are further enshrined in the International Declaration of Human Rights, the Covenant on Economic, Social, and Cultural Rights, CEDAW, the Covenant on Civil and Political Rights, the Convention on the Rights of the Child and the UN Declaration on the Right to Development. The Inter-Agency Standing Committee Policy Statement on Integration of a Gender Perspective in Humanitarian Operations has appealed to address barriers hindering women's effective participation. It has committed to “integrating capacity building of women's organizations in humanitarian response and rehabilitation and recovery phase”, which can strengthen local capacity in humanitarian response [36],[37].

However, Claudia von Braunmühl noted that the UNSCR1325 and the ensuing UN resolutions are restrained by adherence to the liberal and neoliberal paradigm and continue to see women as victims who need to be protected than the agents to be empowered [38]. From Foggy Bottom to Turkey, every possible position was examined within the Ministries of Foreign Affairs (MFA) worldwide to assess if postings of women at certain levels were merely a token appointment in locations of lesser clout or were actual victories of professional accomplishment. Clear hierarchies of power, influence and prestige among positions within the international sector exist. There is a level of “relational and gendered division of labour” as women are seldom appointed to ambassadorial designations [31],[36].

From Japan to Sweden, Brazil to Israel, a wide net was cast by including an extensive geographic spectrum of nations to explore women's rank, numbers, and achievements within international affairs. It concluded that equality and equity could not be achieved by involving women in diplomatic relations; if a woman is not at the table, sitting in the room is of little importance. A seat at the table will be of little consequence if women's perspective and opinions are dismissed as less significant. Compared to other critical priorities such as military politics and economic concerns, it demonstrated that merely counting the increasing number of women doesn't mean that women count in foreign relations [32],[39].

The lack of women at high-level positions or merely a tokenism presentation in the foreign services is disappointing but what is utterly distressing is the almost complete absence or dismissal of women in peace mediations and relevant negotiations on a global scale. Again, the mere presence of women in peace negotiations is insufficient. Moreover, women's exclusion from high-level positions in international finance (central banks, formal and informal networks, regulatory institutions) and the effect of gender-biased knowledge structures can impact humanitarian aid. Given the arguments and themes outlined above clearly, the “glass ceiling” exists for women progressing to senior positions, particularly in diplomacy and peacemaking, impacting the humanitarian aid operations, including issues relevant to SEA. There is a need for explicit inclusion of “gender equality provisions” [32],[40],[41]. To tackle SEA, one cannot neglect the problem-solving pursuit of incremental change from “inside” the world of global governance of humanitarian aid, as it is any more than they can forsake the critical-theoretical push for historical gender-transformative change.

Further, challenges to grabbling SEA are posed by bureaucracies applying gender policy without adequate civil society participation and the struggle to establish global gender standards while confronting conservative political resistance. Gender-aware policies have been reduced to mere administrative routines and checklists. Humanitarian aid missions with the workforce from nations with better records of gender equality, i.e., better records on educated women and their labour force participation, tend to have fewer counts of SEA allegations [32],[42]–[44].

Promoting gender-equality education is essential for violence prevention, and well-placed school programs have shown the potential to influence gender norms and attitudes before they become deeply ingrained. When gender norms are more flexible, most women can enjoy greater independence and power, and the threat of violence against them reduces [45].

Diversity in governments, parliaments and other decision-making positions is necessary. Without it, policies and laws are often made to benefit people who have a seat at the table and authority, which sometimes overlooks the needs of the vulnerable, less powerful population. When it came to handling the crisis caused by the pandemic, female leaders from New Zealand to Germany and Taiwan to Finland took decisive actions in introducing social distancing and quarantine rules. Their empathy, consistent and clear risk communication to citizens based on facts and taking COVID-19 seriously from the start is indicative of sound leadership qualities. Gender equality is a value both men and women can hold; it increases the extent to which the workforce holds it may potentially reduce some of the detrimental manifestations of patriarchy. A holistic approach to lowering SEA by improving gender equality will bring much-needed change and benefits [43],[44].

## 7. Conclusion

Necessary steps must be taken to prevent SEA and hold perpetrators accountable, as the impact of SEA is enormous: on the lives of their survivors, the outcomes of the mission they are part of, and global perception of the legitimacy of the humanitarian project. Women should be allowed to lead from the front; otherwise, true gender equality and issues relevant to gender, including SEA, will be challenging to attain. Besides, we propose more stringent recruitment and training of peacekeepers and aid workers. The prospective humanitarian aid workforce should be evaluated based on their values for gender equality. Survivors of SEA have the right to take back control of and reshape their lives, including the right to sufficient protection and aid; they should be allowed to play an active role in every aspect of crisis response. Moreover, appropriate international human right laws and regulations concerning SEA should form part of the humanitarian workforce training for all international organizations. Finally, strengthening humanitarian diplomacy can play a pivotal role to train the humanitarian workforce on best practices to reduce SEA.
